# Beneficial effects of time and energy restriction diets on the development of experimental acute kidney injury in Rat: Bax/Bcl-2 and histopathological evaluation

**DOI:** 10.1186/s12882-023-03104-6

**Published:** 2023-03-20

**Authors:** Alireza Raji-Amirhasani, Mohammad Khaksari, Zahra Soltani, Shadan Saberi, Maryam Iranpour, Fatemeh Darvishzadeh Mahani, Zahra Hajializadeh, Nazanin Sabet

**Affiliations:** 1 Department of Physiology and Pharmacology, Afzalipour Faculty of Medicine, Kerman University of Medical Sciences, Kerman, Iran; 2 Endocrinology and Metabolism Research Center, Kerman University of Medical Sciences, Kerman, Iran; 3 Student Research Committee, Kerman University of Medical Sciences, Kerman, Iran; 4 Physiology Research Center, Institute of Neuropharmacology, Kerman University of Medical Sciences, Kerman, Iran; 5 Pathology and Stem Cells Research Center, Kerman University of Medical Sciences, Kerman, Iran; 6 Department of Pathology, Afzalipour Faculty of Medicine, Kerman University of Medical Sciences, Kerman, Iran; 7 Cardiovascular Research Center, Kerman University of Medical Sciences, Kerman, Iran

**Keywords:** Acute Kidney Injury, Energy Restriction, Time-restricted Eating, Intermittent Fasting, High-fat Diet, Bax, Bcl-2

## Abstract

People’s lifestyles and, especially, their eating habits affect their health and the functioning of the organs in their bodies, including the kidneys. One’s diet influences the cells’ responses to stressful conditions such as acute kidney injury (AKI). This study aims to determine the preconditioning effects of four different diets: energy restriction (ER) diet, time restriction (TR) eating, intermittent fasting (IF), and high-fat diet (HF) on histopathological indices of the kidney as well as the molecules involved in apoptosis during AKI. Adult male rats underwent ER, TR, IF, and HF diets for eight weeks. Then, AKI was induced, and renal function indices, histopathological indices, and molecules involved in apoptosis were measured. In animals with AKI, urinary albumin excretion, serum urea, creatinine and, Bax/Bcl-2 ratio increased in the kidney, while renal eGFR decreased. ER and TR diets improved renal parameters and prevented an increase in the Bax/Bcl-2 ratio. The IF diet improved renal parameters but had no effect on the Bax/Bcl-2 ratio. On the other hand, the HF diet worsened renal function and increased the Bax/Bcl-2 ratio. Histopathological examination also showed improved kidney conditions in the ER and TR groups and more damage in the HF group. This study demonstrated that ER and TR diets have renoprotective effects on AKI and possibly cause the resistance of kidney cells to damage by reducing the Bax/Bcl-2 ratio and improving apoptotic conditions.

## Introduction

Acute kidney injury (AKI), formerly known as acute renal failure (ARF) is a syndrome in which there is a sudden decline in renal function [1]. AKI is treatable, but mortality is still high (over 50%), so research into effective treatment to accelerate recovery and prevent AKI has received much attention [2]. AKI is associated with a rapid and reversible decrease in Glomerular Filtration Rate (GFR) and increased serum urea and creatinine over hours or days. Damage to the kidney tissue can be caused by several factors, including the kidney’s exposure to harmful substances, renal ischemia, oxidative stress, and inflammation in the kidney or urinary tract obstruction [3, 4].

In the last few decades, the prevalence of obesity has been increasing and has now reached unprecedented levels [5, 6]. Obesity is a disease associated with higher energy intake, which has genetic, metabolic, behavioral, social and environmental causes [7, 8]. Recent research has shown that energy restriction (ER) increases longevity and also minimizes functional impairment and age-related diseases [8–10]. Most studies have shown that ER leads to weight loss, but this weight loss is not sustained over time, and it still has remained as a challenge, because it is difficult to adhere to this method in the long term [11–13]. An ER diet usually includes periods of very low energy intake or fasting [14]. Therefore, ER includes different diets that are somehow associated with reducing energy consumption [14].

Animals subjected to ischemia, which already had an ER diet, were reported to have smaller infarct sizes and milder neurological damages [9, 15, 16]. This diet also caused ischemic tolerance in older rodents [8, 17]. Short-term ER preconditioning has been investigated as a possible strategy to prevent AKI induced by anticancer chemotherapeutic drugs, and it has been shown that the ER diet has protective effects against apoptosis in the kidney of DDP (cisplatin)-treated mice [18]. Short-term dietary interventions can cause resistance to pressure and stress in ischemic models of kidney and liver injury to maintain organ function [18, 19]. Beneficial metabolic effects of ER, possibly through increased SIRT1 expression and autophagy activity, delay apoptosis in renal tubular epithelial cells [18, 20].

Other diets that have been suggested for intervention are fasting or time restriction (TR) diets. In TR, a person can consume calories without restriction, but only for 8 to 10 h a day, but is in the fasting mode for the rest of the day and night [21]. Another type of diet is intermittent or periodic access to food, also known as intermittent fasting (IF), where the individual has access to food every other day [22]. Reducing food consumption (ER, TR, and IF) is different from special diet recommended by a nutritionist, such as the Mediterranean diet, because in the latter type of diet, one will consume certain substances, while in ER, TR, and IF diets, it is not specified which foods are to be eaten or which are forbidden, i.e. one can eat any food [8]. TR and IF have been shown to have effects similar to ER, increasing longevity, attenuating neurodegenerative diseases, cardiovascular disease, and increasing cerebral plasticity [8, 21, 23]. In addition, TR also lowers blood pressure and improves glucose control in humans [8, 24, 25]. Protection of the kidneys against injury by fasting diets (TR and IF) has also been reported [26–28]. It is interesting to know that in contrast to the advantages mentioned for the above three diets, many disadvantages have been reported about one of the today’s diets, namely the high-fat diet (HF) [29–32]. In today’s societies, there have been important changes in the distribution of food and its availability [33, 34]. The expansion of the western lifestyle, which is accompanied by the emergence of food preparation centers and the widespread presence of fast food establishments that produce energy-dense ready meals, has led to high-fat diets becoming common eating habits, and this type of eating is expanding both in urban and rural areas, which is worrying [33, 34]. For example, consuming too many calories [29] and high-fat foods has been shown to cause illness and enhanced vulnerability to diseases [30, 35, 36]. Long-term feeding with high-fat diet causes non-alcoholic fatty liver disease [37]. Also, high fat diet causes oxidative stress and mitochondrial dysfunction in the CNS [35]. Changes in metabolism caused by a high-fat diet aggravate brain dysfunction due to aging and also it accelerates nervous system diseases related to aging [35, 36].

Apoptosis is the programmed cell death seen in multicellular organisms. The intracellular ratio of apoptosis-promoting proteins such as Bax to anti-apoptotic proteins such as Bcl-2 determines the cell’s susceptibility to apoptosis [38, 39]. Apoptosis occurs during AKI in the kidney. In particular, apoptosis in the kidneys is detectable after ischemia, toxin exposure, inflammation, and sepsis [38]. Many of the above injuries occur simultaneously in humans in the intensive care unit [38, 40]. Renal endothelial and epithelial cells are affected by apoptosis in AKI, and this process is one of the leading causes of renal failure in AKI [38]. Previous studies have shown that dietary restrictions and their preconditioning affect apoptosis in organs, including the kidneys. For example, ER has been reported to have a protective effect against kidney damage, the mechanism of which includes exerting anti-apoptotic effects through improving oxidative conditions [18, 41]. Similar anti-apoptotic reports exist for TR [42–44] and IF [8, 45]. On the contrary, several studies have shown that the HF diet can induce apoptosis in the kidney and cause further damage during AKI [46–48].

According to the mentioned studies, manipulating the diet by changing the energy intake or the time of access to food can lead to delays in the onset and progression of diseases and lead to a long healthy life (increasing life expectancy) in most organisms [22, 49], while overeating or under-eating pose potential risks, increasing the incidence of chronic diseases and early death [49, 50]. Among the dietary interventions, the three ER, TR, and IF diets are better known than the other diets, and none of them has been proven entirely superior to the other. Besides, no study has yet compared these diets concerning their effect on AKI-induced damage. In the present study, we evaluated the effects of preconditioning by these diets and compared them with the HF diet regarding their effect on renal function indices, the severity of kidney damage measured through histopathological indices, and the role of molecules involved in apoptosis during experimental AKI.

## Materials and methods

### Animals and their grouping

**Fig. 1:**
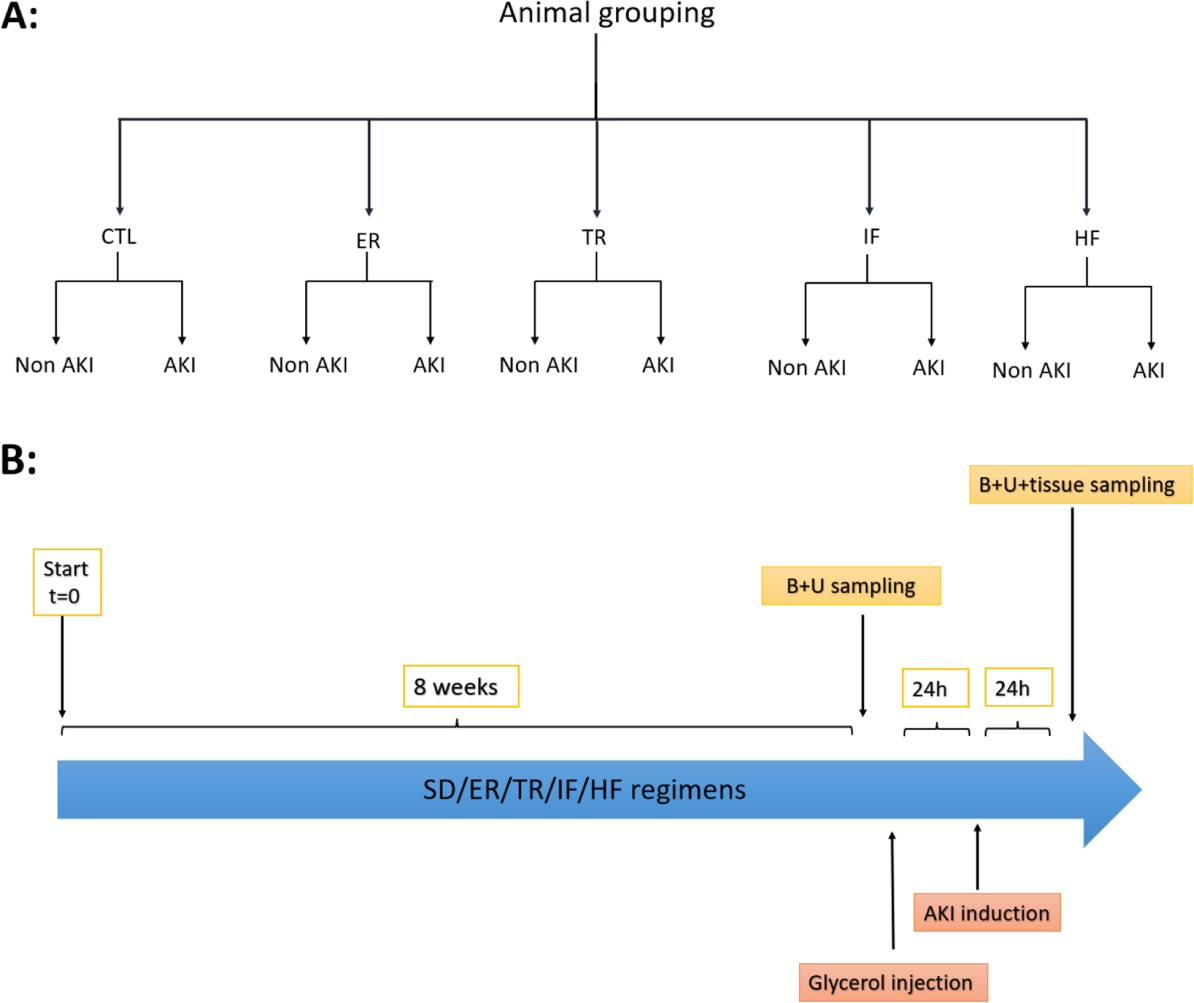
Schematic of **A** animal grouping, **B** representation of the experimental protocol. AKI: acute kidney injury, SD: standard diet or control, ER: energy restriction, TR: time restriction feeding, IF: intermittent fasting, HF: high-fat diet, B: blood, U: urine

In this study, 60 adult male Wistar rats were used. The animals were kept in the natural environment for 12 h in the dark and 12 h in the light at a temperature of 25 °C. This study was conducted in accordance with the regulations of the Ethics Committee of Kerman University of Medical Sciences under the number IR.KMU.REC.1398.270. In this study, animals were divided into five general groups (Fig. 1), and the number of animals in each group was 12:

1. **Control group (CTL):** In this group, animals had free access to food and, after two months, were divided into two subgroups: in one of the subgroups (*n* = 6) renal function indices were measured and after animal sacrifice (under deep anesthesia with intraperitoneal injection of pentobarbital sodium (150 mg/kg)), histopathological markers and molecules involved in apoptosis were measured in kidney tissue. In the other subgroup (*n* = 6), renal function indices were measured one day after the induction of acute kidney injury, and also after animal sacrifice, histopathological markers and molecules involved in apoptosis were measured in kidney tissue [51] (Fig. 1).
2. **Energy restriction group (ER):** Animals in this group consumed 70% of the daily food intake in the control group for two months [52, 53], and after two months, similar to the control group, they were divided into two subgroups (6 rats in each subgroup), and different factors were measured in them (Fig. 1).
3. **Time restriction group (TR):** The animals in this group had free access to food for only 5 h a day for two months [22]. After two months, they were divided into two subgroups (6 rats in each subgroup), and they were treated like the control group (Fig. 1).
4. **Group of animals with intermittent or periodic access to food (IF):** Animals in this group had free access to food every other day for two months (one-day food deprivation and one-day free access to food) [22, 54], and after two months, they were divided into two subgroups (6 rats in each subgroup), and they were treated like the control group (Fig. 1).
5. **High-fat diet (HF):** Animals in this group received a high-fat diet for two months [55], and after two months, they were divided into two subgroups (6 rats in each subgroup) and different factors were measured in them as done in the control group (Fig. 1).

### Measuring the amount of food and applying the energy restriction diet

To calculate the amount of food to be given to the ER group, first, the food intake in the CTL group, which had free access to food, was measured for one week, and then the average daily intake was measured. Then, 70% of the daily intake in the control group, which had free access to food, was calculated and fed to the ER group for two months [53].

### How to create a high-fat diet

A combination of carbohydrates, proteins, and fats was used to make high-fat foods. The high-fat diet consisted of 58% fat, 15% protein, and 27% carbohydrates (Royan institute, Iran), while the standard diet (Pars Animal Feed, Iran) consumed by other groups contained 6% fat, 22% protein, and 72% carbohydrates [56].

### Induction of acute kidney injury

The animals were deprived of water for 24 h, and then a dose of hypertonic glycerol solution (50% dissolved in sterile saline) at 10 ml/kg was injected in equal portions in both lower limbs of the animals intramuscularly (IM) to induce acute kidney injury [3]. With this method, nephropathy developed rapidly, approximately 24 h after glycerol injection. Hypertonic glycerol causes rhabdomyolysis, which eventually leads to myoglobinuria, ischemia, and nephrotoxicity in the kidney [51]. In this study, urea, creatinine, and GFR (estimated by creatinine clearance) were measured to prove the induction of AKI.

### Measurement of serum and urine urea and creatinine

Blood samples were collected from the choroid sinus and immediately centrifuged to isolate the serum one day before and two days after the induction of acute kidney injury to measure the above indices in serum. Also, to measure the same indicators in urine, urine was collected through the metabolic cage one day before and two days after the induction of acute kidney injury. Serum and urine urea and creatinine levels were measured using the ELISA method and according to the kit instructions (MAN, Iran). Values ​​were reported in mg/dl [53, 57].

### Measurement of 24-h albumin excretion

Animals’ urine was collected using a metabolic cage 24 h before and 24 h after the induction of renal failure. Urine albumin concentration was also measured using the ELISA method and according to the kit instructions (MAN, Iran). Then, using the 24-h urine volume, the amount of albumin excreted in 24 h was calculated based on the following formula, and the excretory albumin levels were reported in mg/24 h [53]:

$$\mathrm{Albumin}\;\mathrm{excretion}\;\mathrm{in}\;24\;\mathrm h\:=\:(\mathrm{urine}\;\mathrm{volume}\;\mathrm{in}\;24\;\mathrm h\:\times\:\mathrm{concentration}\;\mathrm{of}\;\mathrm{urine}\;\mathrm{Albumin})$$

### Estimation of GFR

Creatinine clearance was used to calculate estimated GFR (eGFR). Briefly, the animals were kept in a metabolic cage for 24 h, and their urine was collected, both before the induction of acute kidney injury and 24 h after it. Then, using a 24-h urine volume and serum and urine creatinine concentrations, eGFR was calculated based on the following formula [58, 59]:

$$eGFR=\frac{\mathrm{urine}\;\mathrm{volume}\;\mathrm{in}\;24\;\mathrm h\times\mathrm{concentration}\;\mathrm{of}\;\mathrm{urine}\;\mathrm{creatinine}}{\mathrm{concentration}\;\mathrm{of}\;\mathrm{serum}\;\mathrm{creatinine}}$$

Finally, eGFR calculations were corrected for body weight (BW) and expressed as ml/min/100 g BW [59].

### Measurement of histopathological indicators in the kidney

The animals were anesthetized with pentobarbital, and then their kidneys were extracted and placed in 10% formalin buffer. After at least 72 h in formalin, they were washed with water, then dehydrated with alcohol, fixed in paraffin, and finally stained through the hematoxylin and eosin methods and examined microscopically [60]. A professional pathologist (who was blind to the study) determined score of damage in the kidney. The intensity of the injury was from 0 to 3 (no damage = 0, mild injury = 1, moderate injury = 2 and severe injury = 3). The injury parameters that were determined included: cellular vacuolization, congestion, tubular casts, inflammation, cellular necrosis and tubular dilation [61, 62].

### Measurement of Bax and Bcl-2 proteins in kidney tissue

After animals were sacrificed, all the animals’ kidneys were separated and placed in cold physiological saline and then homogenized using a homogenizer and centrifuged for 10 min to prepare supernatant fluid at 1000 rpm. Bax and Bcl-2 levels in kidney tissue were measured using the ELISA method according to the kit instructions (ZellBio, Germany). The values ​​of these indices were reported in ng/mg protein [63, 64].

### Kidney weight to body weight ratio

Animal weights were measured using a digital scale (Gram Precision digital scale, Canada). Also, the kidney of all animals was isolated after sacrifice, the adipose tissue around them was cleared, and the weight of the kidneys was calculated by digital scales [65]. The kidney weight to body weight (KW/BW) ratio, was calculated by dividing left kidney weight by body weight and then was converted to percent.

### Method of calculating and analyzing data

Two-way ANOVA test followed by Tukey’s test was used to compare the quantitative variables between the test groups if the assumptions were observed (normal data distribution), and the Kruskal–Wallis test was applied in case the assumptions were not fulfilled. A significance level of 0.05 was considered, and statistical analyses were performed using GraphPad Prism 8.

## Results

### Effects of diets on renal parameters in animals with and without AKI

We investigated the effects of AKI and/or diets on serum urea and creatinine in the rats. Two way ANOVA showed a significant interaction between AKI and diets for serum urea [F (4,50) = 39.58, *P* < 0.001] and serum creatinine [F (4,50) = 32.03, *P* < 0.001]. In addition two-way ANOVA revealed that the main effect of AKI for serum urea [F (1,50) = 1276, *P* < 0.001] and serum creatinine [F (1,50) = 1011, *P* < 0.001]), and also the main effect of diets for serum urea [F (4,50) = 24.00, *P* < 0.001] and serum creatinine [F (4,50) = 32.98, *P* < 0.001] were significant. The post hoc Tukey test revealed, in animals without AKI, serum urea and creatinine were not significantly different from the control group in any of the groups. Twenty-four hours after AKI induction, serum urea and creatinine increased significantly in all groups with AKI, when compared to groups with similar diets but without AKI (*P* < 0.001). However, the increase in urea and creatinine in TR after AKI induction was smaller than their increase in the ER (*P* < 0.01), CTL, and HF (*P* < 0.001) groups and smaller than the IF group for creatinine (*P* < 0.001). In animals with AKI, urea showed a smaller increase in the ER and IF groups than CTL and HF groups (*P* < 0.001). Also, the increase in serum creatinine after AKI in the ER group was smaller than in the IF (*P* < 0.01), CTL, and HF (*P* < 0.001) groups. In the IF group, the increase in serum creatinine after AKI was smaller than in the CTL (*P* < 0.05) and HF (*P* < 0.001) groups. While Serum urea and creatinine levels after AKI in the HF group were higher than in the CTL group (*P* < 0.001 and *P* < 0.05, respectively) (Table 1).

**Table 1:** The effects of diets on renal parameters in animals with and without AKI

	**Without AKI**					**With AKI**				
**Groups**	CTL	ER	TR	IF	HF	CTL	ER	TR	IF	HF
**Parameters**										
Serum Urea (mg/dl)	70.83 ± 3.37	97.01 ± 5.35	83.01 ± 6.02	67.02 ± 4.85	60.33 ± 4.18	312 ± 10.51^***^	253.66 ± 6.88^†††^	204 ± 10.7^###, ##^	241 ± 15.22^^^^^	383.5 ± 14.38^&&&^
Serum Creatinine (mg/dl)	0.45 ± 0.03	0.57 ± 0.01	0.53 ± 0.02	0.54 ± 0.03	0.61 ± 0.07	3.38 ± 0.14^***^	2.3 ± 0.15 ^†††, ††^	1.76 ± 0.1 ^###, ##^	2.86 ± 0.07^^^^, ^^	3.88 ± 0.17^&^
Urine Albumin excretion (mg/24 h)	0.51 ± 0.17	0.58 ± 0.15	0.41 ± 0.15	0.5 ± 0.18	0.75 ± 0.17	28.16 ± 1.92^***^	24.5 ± 1.97^††^	16.66 ± 0.8^###, #^	22.3 ± 0.66^^^^, ^^	31.83 ± 2.5
eGFR (ml/min/100 g bw)	0.52 ± 0.01	0.48 ± 0.02	0.52 ± 0.02	0.52 ± 0.02	0.49 ± 0.02	0.1 ± 0.007^***^	0.18 ± 0.004^†^	0.26 ± 0.008^###, #^	0.11 ± 0.01	0.1 ± 0.004
Body weight (g)	244 ± 4.84	193 ± 3.43^ΩΩΩ^	261 ± 5.13	238 ± 5.32	285 ± 5.49^ΦΦΦ^	245 ± 4.43	192 ± 2.34	257 ± 4.11	240 ± 4.62	286 ± 5.07
Kidney weight (g)	1.008 ± 0.024	0.74 ± 0.023^ΩΩΩ^	1.001 ± 0.042	0.916 ± 0.036	1.188 ± 0.029^ΦΦΦ^	1.443 ± 0.031^***^	1.051 ± 0.035	1.373 ± 0.034	1.361 ± 0.027	1.776 ± 0.043
KW/BW ratio	0.41 ± 0.013	0.38 ± 0.008	0.38 ± 0.016	0.38 ± 0.015	0.41 ± 0.009	0.59 ± 0.01^***^	0.54 ± 0.016^††^	0.53 ± 0.014^##^	0.57 ± 0.015	0.62 ± 0.015

Experimental groups (*n* = 6). Findings are reported based on Mean ± SEM

*CTL* control, *ER* energy restriction, *TR* time restriction, *IF* intermittent fasting, *HF* high-fat diet, *eGFR* estimated glomerular filtration rate, *KW/BW ratio* Kidney weight to body weight ratio, *AKI* acute kidney injury

^***^ *P* < 0.001* VS*. CTL without AKI for all parameters

Serum urea in animals with AKI: ^†††^ *P* < 0.001 *VS.* CTL and HF. ^##^ *P* < 0.01* VS.* ER. ^###^ *P* < 0.001 *VS.* CTL and HF. ^^^^^ *P* < 0.001 *VS.* CTL and HF. ^&&&^ *P* < 0.001 *VS.* CTL

Serum creatinine in animals with AKI: ^††^ *P* < 0.01* VS.* IF. ^†††^ *P* < 0.001* VS.* CTL and HF. ^##^ *P* < 0.01* VS.* ER. ^###^ *P*< 0.001* VS.* CTL, IF, and HF. ^^^ *P* < 0.05* VS.* CTL. ^^^^^ *P*< 0.001* VS.* HF. ^&^ *P* < 0.05 *VS.* CTL

Urinary albumin excretion in animals with AKI: ^††^ *P* < 0.01* VS.* HF. ^#^ *P* < 0.05* VS.* IF. ^###^ *P* < 0.001* VS.* CTL, ER, and HF. ^^^ *P* < 0.05* VS.* CTL, ^^^^^ *P* < 0.001* VS.* HF

GFR in animals with AKI: ^†^ *P* < 0.05* VS.* CTL, IF, and HF. ^#^ *P* < 0.05* VS.* ER. ^###^ *P* < 0.001* VS.* IF, CTL, and HF

Body weight and Kidney weight in animals without AKI: ^ΩΩΩ^ *P* < 0.001* VS*. CTL. ^ΦΦΦ^ *P* < 0.001* VS*. CTL

KW/BW ratio in animals with AKI: ^††^ *P* < 0.01* VS.* HF. ^##^ *P* < 0.01* VS.* HF

Two way ANOVA showed a significant interaction between AKI and diets for urinary albumin excretion [F (4,50) = 10.58, *P* < 0.001] and eGFR [F (4,50) = 10.39, *P* < 0.001]. In addition two-way ANOVA revealed that the main effect of AKI for urinary albumin excretion [F (1,50) = 963.1, *P* < 0.001] and eGFR [F (1,50) = 1213, *P* < 0.001], and also the main effect of diets for urinary albumin excretion [F (4,50) = 11.41, *P* < 0.001] and eGFR [F (4,50) = 10.57, *P* < 0.001] were significant. The post hoc Tukey test revealed that, none of the diets had a significant effect in urinary albumin excretion and eGFR in animals without AKI. Within 24 h after induction of AKI, urinary albumin excretion increased significantly in all groups with AKI compared to similar groups without AKI (*P* < 0.001). However, this increase in urinary albumin excretion was smaller in the TR group than in the IF (*P* < 0.05), CTL, ER, and HF (*P* < 0.001) groups. In addition, in animals with AKI, the increase in urinary albumin excretion was lower in the IF group than in the CTL (*P* < 0.05) and HF (*P* < 0.001) groups and smaller in the ER group than in the HF group (*P* < 0.01). In animals with AKI, eGFR showed a significant decrease in all groups compared to similar groups before AKI (*P* < 0.001). The decrease in eGFR after AKI was smaller in the TR group than in the ER (*P* < 0.05), CTL, IF, and HF groups (*P* < 0.001) and in the ER group compared to the CTL, IF, and HF groups (*P* < 0.05) (Table 1).

Two way ANOVA showed that there was no significant interaction between AKI and diets for body weight [F (4,50) = 0.08078, *P* = 0.9879], but there was significant interaction for kidney weight [F (4,50) = 4.694, *P* = 0.0027]. In addition two-way ANOVA revealed that the main effect of AKI was not significant for body weight [F (1,50) = 0.0005306, *P* = 0.9817], but was significant for kidney weight [F (1,50) = 406.9, *P* < 0.001]. Also the main effect of diets was significant for both body weight [F (4,50) = 110.0, *P* < 0.001] and kidney weight [F (4,50) = 77.29, *P* < 0.001]. The post hoc Tukey test revealed that after two months of dieting, body and kidney weight decreased in the ER group and increased in the HF group compared to the CTL (*P* < *0.001*) and didn’t change in TR and IF groups. There was a significant increase in kidney weight in all groups with AKI compared to similar groups without AKI (*P* < 0.001). We used the KW/BW ratio to compare renal edema among the groups. Two way ANOVA showed that there was no significant interaction between AKI and diets for KW/BW ratio [F (4,50) = 1.076, *P* = 0.3783], but the main effects of AKI and diets were significant for KW/BW ratio [F (1,50) = 408.8, *P* < 0.001] and [F (4,50) = 6.977, *P* < 0.001], respectively. The post hoc Tukey test revealed that in animals without AKI, the KW/BW ratio did not differ in study groups, but this ratio increased in animals with AKI in all groups compared to similar groups without AKI (*P* < *0.001*). The increase of this index in the ER and TR groups was less than HF group (*P* < *0.01*). Although the increase in this index after injury in ER and TR groups was less than CTL group, it was not statistically significant (Table 1).

### Comparison of histopathological indicators of the kidney in animals with and without AKI in the presence of different diets

**Fig. 2:**
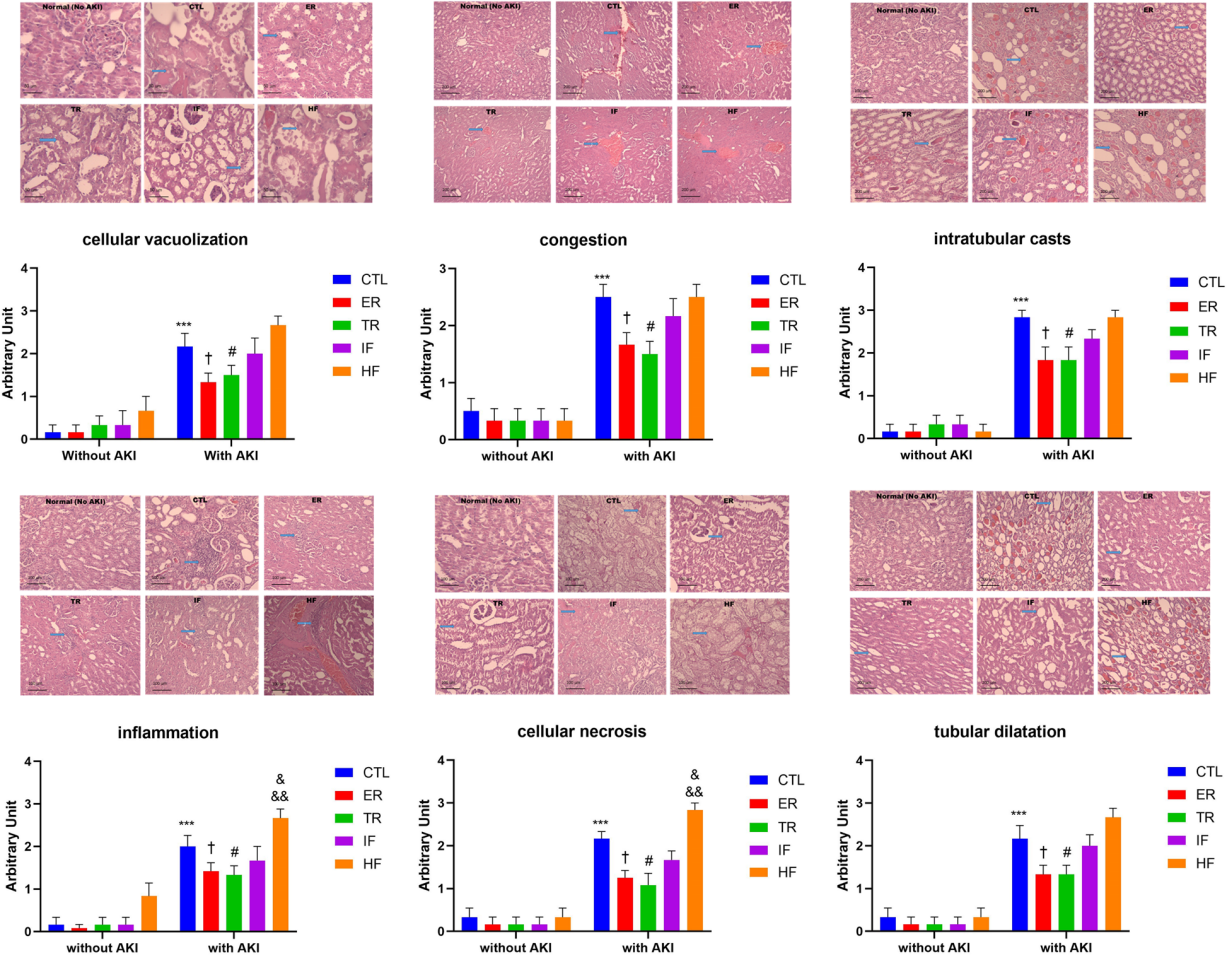
Renal histopathological changes after AKI in experimental groups (*n* = 6). Findings are reported based on Mean ± SEM (magnification × 400 for vacuolization and × 100 for other indices). ^***^ *P* < 0.001* VS.* CTL group without AKI. ^†^ *P* < 0.05* VS.* CTL with AKI. ^#^ *P* < 0.05 *VS.* CTL with AKI. ^&^ *P* < 0.05* VS.* CTL with AKI. ^&&^ *P* < 0.01* VS.* ER, TR and IF with AKI. CTL: control, ER: energy restriction, TR: time restriction, IF: intermittent fasting, HF: high-fat diet, AKI: acute kidney injury

We investigated the effects of AKI and/or diets on histopathological indicators of the kidney. Two way ANOVA showed a significant interaction between AKI and diets for cell vacuolization, congestion, intratubular cast, tubular dilatation, inflammation, and necrosis [F (4,50) = 2.857, *P* = 0.0329], [F (4,50) = 2.863, *P* = 0.0326], [F (4,50) = 3.720, *P* = 0.01], [F (4,50) = 3.754, *P* = 0.0095], [F (4, 50) = 4.600, *P* = 0.0031] and [F (4, 50) = 6.560, *P* = 0.0003], respectively. In addition two-way ANOVA revealed that the main effect of AKI was significant for cell vacuolization, congestion, intratubular cast, tubular dilatation, inflammation, and necrosis [F (1,50) = 102.9, *P* < 0.001], [F (1,50) = 128.9, *P* < 0.001], [F (1,50) = 230.2, *P* < 0.001], [F (1,50) = 168.4, *P* < 0.001], [F (1,50) = 218.1, *P* < 0.001] and [F (1,50) = 228.9, *P* < 0.001], respectively. Also the main effect of diets was significant for cell vacuolization, congestion, intratubular cast, tubular dilatation, inflammation, and necrosis [F (4,50) = 5.089, *P* = 0.0016], [F (4,50) = 3.712, *P* = 0.0101], [F (4,50) = 2.792, *P* = 0.0360], [F (4,50) = 6.125, *P* < 0.001], [F (4,50) = 14.98, *P* < 0.001] and [F (4,50) = 10.23, *P* < 0.001], respectively. The post hoc Tukey test revealed that, the rates of cell vacuolization, congestion, intratubular cast, tubular dilatation, inflammation, and necrosis in the kidney, did not differ in groups without AKI, but these indices increased in animals with AKI, when compared to groups with similar diets but without AKI (*P* < 0.001); however, these indices in the ER and TR groups showed a smaller increase compared to the CTL group (*P* < 0.05). In other words, ER and TR diets could prevent the increase of injury symptoms after AKI to some extent. Also, the rate of inflammation and necrosis after AKI in the HF group was higher than in all the other groups (*P* < 0.05 for CTL and *P* < 0.01 for ER, TR and IF) (Fig. 2).

### Comparison of renal Bax levels in experimental groups

**Fig. 3:**
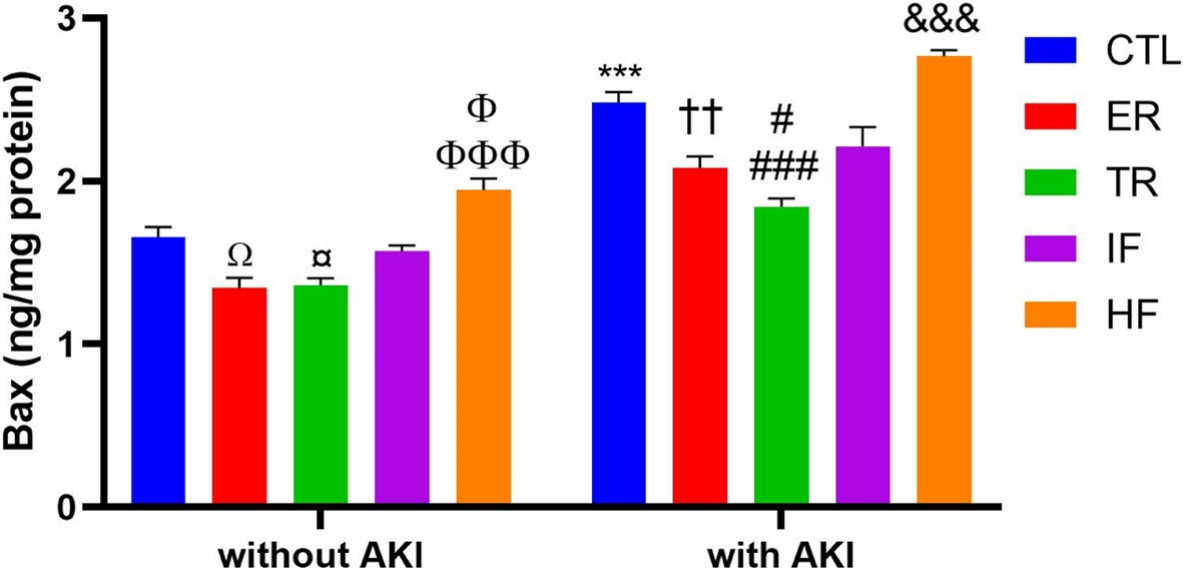
Renal Bax level (ng/mg protein) in experimental groups (*n* = 6). Findings are reported based on Mean ± SEM. ^Ω^ *P* < 0.05* VS.* CTL without AKI, ^¤^ *P* < 0.05* VS.* CTL without AKI. ^ΦΦΦ^ *P* < 0.001 *VS.* ER and TR without AKI. ^Φ^ *P* < 0.05 *VS.* IF without AKI. ^***^ *P* < 0.001* VS.* CTL without AKI. ^††^ *P* < 0.01 *VS.* CTL with AKI. ^###^ *P* < 0.001* VS.* CTL with AKI. ^#^ *P* < 0.05 *VS.* IF with AKI. ^&&&^ *P* < 0.001* VS.* ER, TR and IF with AKI. CTL: control, ER: energy restriction, TR: time restriction, IF: intermittent fasting, HF: high-fat diet, AKI: acute kidney injury

Two way ANOVA showed that there was no significant interaction between AKI and diets for Bax levels in the kidney [F (4,50) = 2.451, *P* = 0.0580], but the main effects of AKI and diets were significant for Bax levels [F (1,50) = 291.5, *P* < 0.001] and [F (4,50) = 42.36, *P* < 0.001], respectively.The post hoc Tukey test revealed that in animals without AKI, ER, and TR diets decreased renal Bax compared to the CTL group (*P* < 0.05), while HF increased this index compared to the ER, TR (*P* < 0.001), and IF (*P* < 0.05) diets. In animals with AKI, the amount of this protein increased in the kidney of all groups compared to similar groups without AKI (*P* < 0.001), but this increase was smaller in ER and TR groups compared to the CTL group (*P* < 0.01 and *P* < 0.001, respectively). This index was also lower than IF in the TR group after AKI (*P* < 0.05). In addition, the increase in Bax after AKI in the HF group was greater than in the ER, TR, and IF groups (*P* < 0.001) (Fig. 3).

### Comparison of renal Bcl-2 levels in experimental groups

**Fig. 4:**
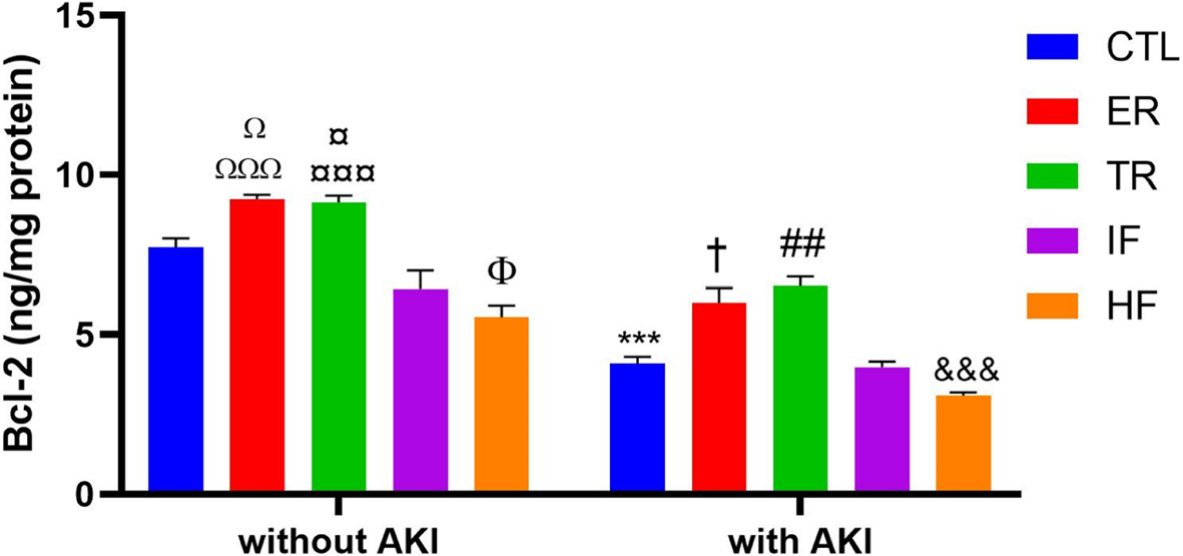
Renal Bcl-2 level (ng/mg protein) in experimental groups (*n* = 6). Findings are reported based on Mean ± SEM. ^Ω^ *P* < 0. 05* VS.* CTL without AKI. ^ΩΩΩ^ *P* < 0. 001* VS.* IF and HF without AKI. ^¤^ *P* < 0.05* VS.* CTL without AKI. ^¤¤¤^ *P* < 0.001* VS.* IF and HF without AKI. ^Φ^ *P* < 0.05* VS.* CTL without AKI. ^***^ *P* < 0.001* VS.* CTL without AKI. ^†^ *P* < 0.05* VS.* CTL and IF with AKI. ^##^ *P* < 0.01* VS.* CTL and IF with AKI. ^&&&^ *P* < 0.001, compared to ER and TR with AKI. CTL: control, ER: energy restriction, TR: time restriction, IF: intermittent fasting, HF: high-fat diet, AKI: acute kidney injury

Two way ANOVA showed that there was no significant interaction between AKI and diets for Bcl-2 levels in the kidney [F (4,50) = 1.388, P = 0.2517], but the main effects of AKI and diets were significant for Bcl-2 levels [F (1,50) = 205.9, *P* < 0.001] and [F (4,50) = 46.20, P < 0.001], respectively. The post hoc Tukey test revealed that in animals without AKI, the ER and TR diets increased renal Bcl-2 compared to the CTL (*P* < 0.05), IF and HF groups (*P* < 0.001), and the HF diet decreased this protein in comparison with the CTL group (*P* < 0.05) (Fig. 4). In animals with AKI, the amount of this protein decreased in the kidney compared to similar groups without AKI (*P* < 0.001), but this decrease in ER and TR groups compared to the CTL, IF (*P* < 0.05, *P* < 0.01, respectively), and HF (*P* < 0.001) groups was smaller (Fig. 4).

### Comparison of renal Bax/Bcl-2 ratio in experimental groups

**Fig. 5:**
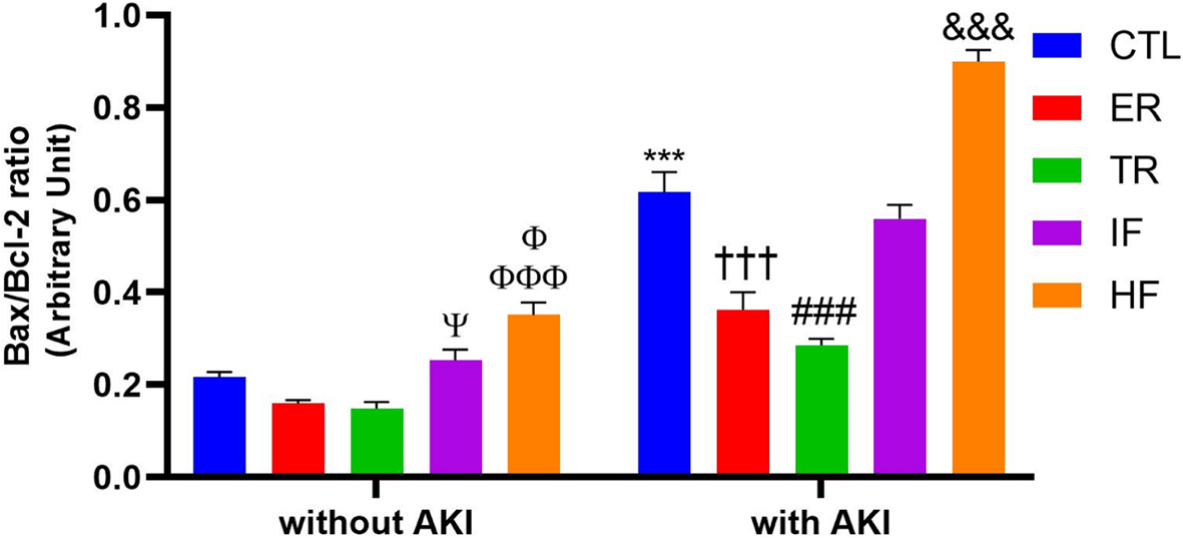
Renal Bax/Bcl-2 ratio in experimental groups (*n* = 6). Findings are reported based on Mean ± SEM. ^Ψ^ *P* < 0.05* VS.* ER and TR without AKI. ^Φ^ *P* < 0.05* VS.* CTL without AKI. ^ΦΦΦ^ *P* < 0.001 *VS.* ER and TR without AKI. ^***^ *P* < 0.001* VS.* CTL without AKI. ^†††^ *P* < 0.001* VS.* CTL, IF and HF with AKI. ^###^ *P* < 0.001* VS.* CTL, IF, and HF with AKI. ^&&&^ *P* < 0.001* VS.* CTL and IF with AKI. CTL: control, ER: energy restriction, TR: time restriction, IF: intermittent fasting, HF: high-fat diet, AKI: acute kidney injury

Two way ANOVA showed a significant interaction between AKI and diets for Bax/Bcl-2 ratio in the kidney [F (4,50) = 19.48, *P* < 0.001]. In addition two-way ANOVA revealed that the main effects of AKI and diets were significant for Bax/Bcl-2 ratio in the kidney [F (1,50) = 372.9, *P* < 0.001] and [F (4,50) = 75.42, *P* < 0.001], respectively. The post hoc Tukey test revealed that in animals without AKI, the renal Bax/Bcl-2 ratio in the IF group was higher than in the ER and TR groups (*P* < 0.05), but it was not significantly different from the CTL group. Also in animals without AKI, this index in the HF group was higher than in the ER, TR (*P* < 0.001), and CTL (*P* < 0.05) groups. In animals with AKI, this ratio increased in all groups compared to similar groups without AKI (*P* < 0.001), but this increase was smaller in the ER and TR groups than in the other groups (*P* < 0.001). Also, this index was higher after AKI in the HF group than in the CTL and IF groups (*P* < 0.001) (Fig. 5).

## Discussion

The results of our study, which was conducted to investigate the effects of four different diets, ER, TR, IF, and HF, on biochemical and histopathological indices and molecules affecting apoptosis in the kidney, showed that the application of these diets had different effects on the level and severity of damage symptoms before and after experimental AKI. The main findings of this study are 1- After AKI, the ER, TR, and IF diets prevented an increase in serum urea and creatinine, while HF increased them. 2- After AKI, the TR, and IF groups showed decreased urinary albumin excretion. 3- eGFR, after AKI, showed a smaller decrease in the ER and TR groups. 4- Histopathological indices after AKI showed abnormal kidney status, while these indices in the ER and TR groups showed better kidney health status. 5- Before AKI, the Bax/Bcl-2 ratio increased in the HF group, indicating apoptosis. After AKI, this ratio increased in all groups, although this increase was smaller in the ER and TR groups and greater in the HF group, indicating a worsening of apoptotic conditions in the latter group.

In the present study, after AKI, renal injury indices (serum creatinine and urea, urinary albumin excretion, and eGFR) changed, while the ER, TR, and IF diets prevented the increase of serum creatinine and urea and urinary albumin excretion and a decrease in eGFR. However, the HF diet did not have a positive effect but worsened the injury indices. Consistent with our results, many studies have shown that different models of dietary restriction are effective in protecting the kidney against AKI: normalization of creatinine levels and reduction of urea and protein excretion in urine after AKI by ER [18, 66], protection of the kidney against I/R damage by fasting [26], increased renal resistance to damage by ER and TR [27], and renoprotective effects of IF in diabetes [28]. Also, previous work by our research team has shown that ER and TR diets can prevent further damage during AKI [67]. Possible mechanism(s) of the positive effects of these diets in reducing kidney damage are: improvement of mitochondrial function [26], effect on various transcription factors including FOXO3, HNF4A, HMGA1, and HSF1 [27], increased expression of SIRT1, SIRT3, and activation SOD2 [18, 67, 68], decreased TGF-β1 and mitochondrial superoxide production and increased GSH concentration [67, 69].

In line with the results of our study, it has been reported that a high-fat diet makes the kidneys more vulnerable when exposed to stress [70]. This diet also causes inflammation in the kidney [71] and further kidney damage during ischemia–reperfusion [72]. Recently, Jeon et al. reported that the HF diet increased susceptibility to ischemic AKI by increasing proinflammatory cytokines [73]. Another study has shown that a high-fat diet causes damage to the kidney structure, including dilation of the tubules and Bowman’s capsule [74]. Further reasons for the vulnerability caused by this type of diet, disruption of the oxidative balance system and an increase in proinflammatory cytokines such as TNF-α, IFN-γ, MCP-1, IL1β, and inflammatory conditions, decreased renal VEGF, increased intra-renal CD8 T cells, renin-angiotensin system imbalance, and insulin resistance, have been reported [73–77].

Histopathological results in animal with AKI indicate that the symptoms of injury are fewer in the ER and TR groups and more in the HF group than in the other groups. Consistent with the results of our study, histopathological studies of animals on the ER diet have reduced renal damage due to aging by reducing cast formation and renal inflammation [78, 79]. A study conducted by Ning et al. (2013) showed that the ER diet prevents dilatation of tubules during cisplatin-induced AKI [18]. Recent studies have also reported that ER reduces kidney damage, such as vacuolization, during AKI [80, 81].

Jongbloed et al. observed that fasting reduced necrosis symptoms in histopathological observations in AKI [82]. It has also been reported that the TR diet can improve histological parameters in other tissues, including the liver [83]. Contrary to our results, it has been reported that fasting does not reduce necrosis symptoms after renal ischemia–reperfusion [84]. The inconsistency between these results and ours is probably the short fasting or energy restriction periods, short-term follow-up, and the AKI model.

There are reports about the harmful effects of HF on the kidneys and its higher vulnerability when exposed to stress [70], causing inflammation in the kidneys [71] and further kidney damage during ischemia–reperfusion [72]. Recently, Jeon et al. (2021) reported that HF ​​could predispose kidneys to AKI, and histologically, HF exacerbated necrosis in the renal tissue [73]. In another study, Eddy et al. reported that HF ​​caused inflammation and eventually interstitial fibrosis of kidney tissue [85]. Also, vacuolization of tubular cells [48], dilatation of tubules, further damage to glomeruli and proximal tubules [74, 86], severe inflammation, destruction of the basement membrane of epithelial cells of renal tubules, and loss of tubular epithelium [87] resulting from an HF diet have been reported in other histopathological studies.

It was stated above that diets were effective in reducing apoptosis. To determine the effect of different diets on apoptosis, this study also measured Bax, Bcl-2, and the ratio of these two proteins (Bax/Bcl-2) in the kidney. The results showed that in animal without AKI, the ER and TR diets decreased Bax and increased Bcl-2 and, conversely, the HF diet had the opposite effect. However, the ratio of these two proteins remained constant in the ER and TR groups and increased in the HF group. After AKI, renal Bax levels increased, Bcl-2 decreased, and the ratio of the two also increased, indicating apoptosis in the kidney. The ER and TR diets reduced apoptosis by preventing changes in these two proteins and their proportions. However, the condition worsened in the HF diet, and the Bax/Bcl-2 ratio increased, indicating an increase in renal apoptosis in this group after AKI.

Consistent with our results, it has been reported that the ER diet has a protective role against AKI by reducing the Bax/Bcl-2 ratio, ultimately reducing apoptosis [18, 66], and it has also been reported to prevent the increase in renal Bax and decrease in Bcl-2 and thus inhibit apoptosis in elder animals [41]. The effect of ER on apoptosis has also been reported for other tissues: decreased Bax/Bcl-2 ratio in the liver and delayed aging [88], increased Bcl-2 and Bcl-2/Bax ratio, and suppression of neuroapoptosis in the brain after brain injury [89], increased Bcl-2 and Bcl-2/Bax ratio in the heart, and protection of the heart against ischemia [90].

Regarding the effects of diets other than ER, it has been reported that the IF diet has a neuroprotective effect on acute spinal cord injury by reducing the Bax/Bcl-2 ratio [45]. The TR diet has been shown to have protective effects on the heart by attenuating apoptosis, regulating the autophagy process, and preserving cells [42]. Another study found that preoperative fasting was protective against renal IRI (ischemic reperfusion injury) by inhibiting apoptosis [91]. Ischemic apoptosis in the kidney is the leading cause of injury, so inhibition of the apoptotic process prevents inflammation and subsequent kidney damage [26]. Numerous studies have also reported that the HF diet can increase Bax, decrease Bcl-2, and ultimately induce apoptosis in the kidney [46–48].

It has already been reported that ER and TR diets increase the survival of cells against damage by increasing Sirt1 and improving the antioxidant system, but IF diet had no effect [67]. It can be said that among these diets, maybe TR can be associated with less difficulty for humans and is recommended. The other two diets (ER and IF), perhaps due to their limitations and difficulty, should be entered into human studies more carefully, and the individual’s health status should also be taken into account so that such diets do not cause harm.

The limitations of this study include: the small number of animals in each group and the limitations of using creatinine clearance as an estimate of GFR.

## Conclusion

Of the four diets used in the present study, the ER and TR diets were more effective in reducing kidney damage during AKI, although the IF diet also reduced serum urea, creatinine, and urinary albumin excretion. The renoprotective mechanism of the ER and TR diets appears to be similar, and histopathological results confirm it. Both diets also reduced the Bax/Bcl-2 ratio, possibly reducing apoptosis. On the other hand, the effects of the HF diet on histopathological indices and Bax/Bcl-2 ratio were opposite the other three diets, i.e., it worsen AKI. This study emphasizes the role of diets in pathophysiological conditions and suggests that future studies aimed at more prolonged periods of fasting or energy restriction and more extended follow-up periods may shed more light on the present findings.

## Data Availability

The datasets used during the current study available from the corresponding author on reasonable request.
